# Delayed presentation of isolated grade III pancreatic injury—a case report

**DOI:** 10.1093/jscr/rjad573

**Published:** 2023-10-17

**Authors:** Maria João Ferreira, Gabriel Gallardo, Emanuel Vigia, Edite Filipe, Hugo Pinto Marques

**Affiliations:** Cirurgia Geral, Centro Hospitalar Tondela Viseu, 3504-509 Viseu, Portugal; HPB unit, Hospital Curry Cabral, Centro Hospitalar Lisboa Central, 2770-049 Lisboa, Portugal; HPB unit, Hospital Curry Cabral, Centro Hospitalar Lisboa Central, 2770-049 Lisboa, Portugal; HPB unit, Hospital Curry Cabral, Centro Hospitalar Lisboa Central, 2770-049 Lisboa, Portugal; HPB unit, Hospital Curry Cabral, Centro Hospitalar Lisboa Central, 2770-049 Lisboa, Portugal

**Keywords:** trauma, pancreatic surgery, case report

## Abstract

Because of their vague and subtle indications and symptoms, pancreatic injuries are frequently misdiagnosed. It’s crucial to have a high level of clinical suspicion. The presence of other organ solid lesions and vascular injuries, as well as the patient’s hemodynamic condition, will determine how these injuries are treated. A surgical approach is mandatory when a ductal disruption occurs. The case of a 32-year-old man who experienced an upper abdominal blunt trauma is presented. He was admitted to our hospital with an acute abdomen 48 hours later. A complete transection of the major pancreatic duct was discovered during surgical investigation, and a distal pancreatectomy with en bloc splenectomy was performed. Even in a delayed context, distal pancreatectomy can be safely performed and is the best option.

## Introduction

Pancreatic injuries are infrequent when compared with other solid organ lesions [1]. Because the pancreas is located in the retroperitoneal cavity, there are often no evident early signs or symptoms [2], so a high level of clinical suspicion is crucial. The pancreatic damage is frequently coupled with other traumatic injuries [3], which explains why pancreatic injuries have such a high rate of complications and mortality.

The most common method of pancreatic injury is blunt direct trauma to the abdomen, which can occur in car accidents, motorcycle accidents, or bicycle accidents.

In recent years, some clinical reports and database studies from high volume centers have helped to establish the needed pancreatic damage management consensus guidelines [2, 4].

A grade III pancreatic injury, defined as a distal transection or parenchymal injury with duct injury on the American Association for Surgery of Trauma’s (AAST) organ injury scale, is one of the most common injuries.

We discuss here the example of a blunt pancreatic trauma that was initially assessed at a local trauma hospital before being referred to a high-volume pancreatic center following the initial approach in another institution.

## Case presentation

We discuss the case of a 32-year-old man who was crushed against a bicycle handlebar and sustained extensive traumatic abdominal damage. He had no relevant past medical or familial records. After the accident, the patient attended the emergency room (ER) immediately. During the initial exam at his local hospital, the patient was hemodynamically stable and had no substantial abdominal pain. Ultrasonography revealed no free fluid, and blood samples revealed no substantial changes. The patient was discharged. He returned to the same ER 12 hours later with ongoing upper abdominal pain. He was still hemodynamically stable, although he was presenting abdominal tenderness at the moment. White blood cell count was 15.8 x 10^9^, hemoglobin was 16.4 g/dl, C-reactive protein (CRP) was 220.2 mg/L, and amylase was 1784 U/L in blood samples.

**Figure 1:**
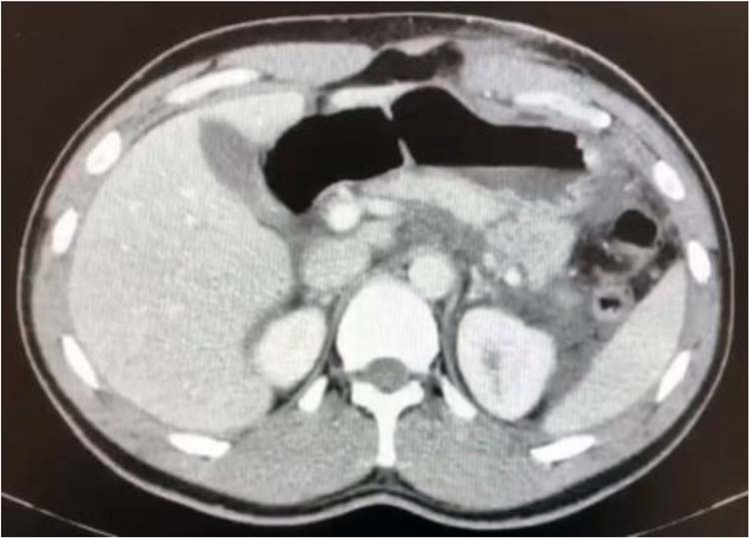
Transection of the pancreatic parenchyma throughout the distal body area (suggesting ductal damage) with a significant fluid collection.

As shown in Fig. 1, an abdominal CT (computerized tomography) scan indicated a full pancreatic parenchyma transection along the body-to-tail transition. There was also a significant amount of free peritoneal fluid.

**Figure 2:**
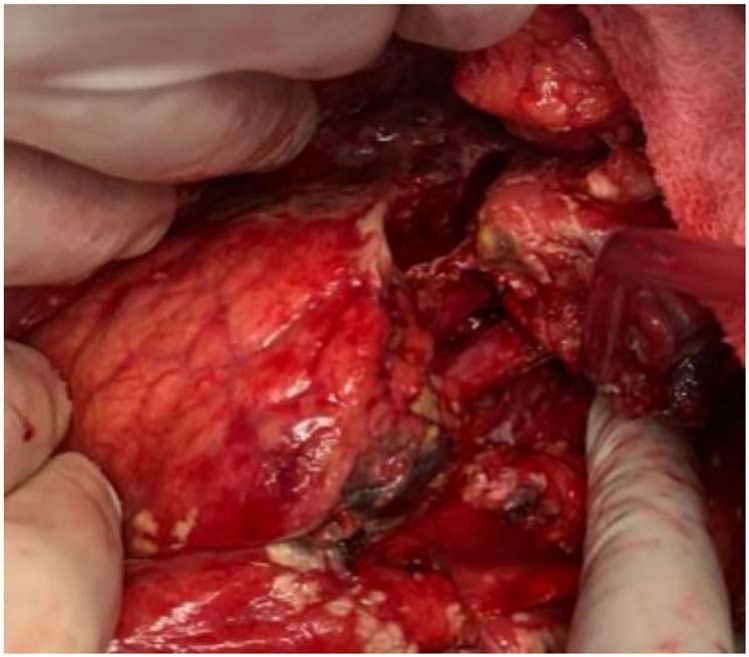
Pancreatic body complete transection (thick arrow—splenic artery; thin arrow—splenic vein).

**Figure 3:**
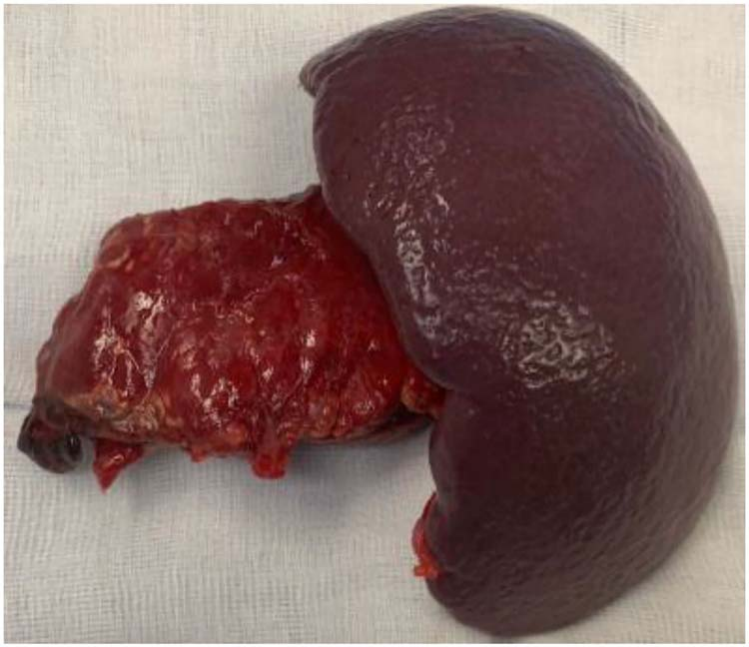
Distal pancreatectomy with en bloc splenectomy specimen.

At that time, the patient was referred to our hepatopancreatobiliary (HPB) unit for definitive treatment based on this clinical data. The patient remained stable throughout first evaluation at our institution, although acute abdomen signs were detected, prompting an abdominal exploration. A grade III AAST pancreatic body damage with ductal transection was discovered during surgery, along with 500 mL of hemoperitoneum. After thorough lavage, since the patient remained stable, a distal pancreatectomy with splenectomy was performed and an accessory spleen remained in place. Figure 2 shows the body pancreatic transection, with just the splenic vessels holding the tail together. Near the pancreatic resection line, two suction drains were placed. The distal pancreatectomy with en bloc splenectomy specimen is shown in Fig. 3.

**Figure 4:**
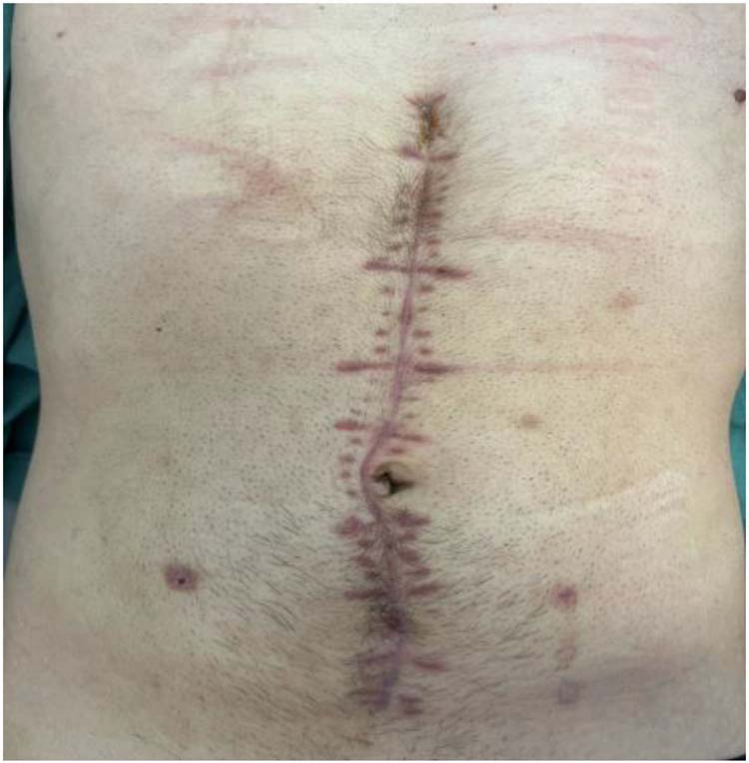
Patient abdominal wound—6 weeks after surgery.

The initial postoperative course was favorable and the patient was discharge 13 days after the surgical procedure, after he tested positive for SARS-COV 2 virus. He developed no symptoms related to the viral infection. He returned to the ER on the 21st day following surgery with purulent leakage from the abdominal wound. A CT showed an intra-abdominal fluid collection that was successfully managed after a week course of intra-venous antibiotic. Figure 4 shows the patient abdominal wound 6 weeks after the surgery. The patient returned to his normal lifestyle shortly after.

## Discussion

Pancreatic trauma injuries are infrequent, accounting for <5% of all abdominal injuries [5]. Pancreatic injuries can cause significant morbidity and possibly death if not discovered early [2]. This particular clinical example demonstrates the need of considering pancreatic lesions when treating upper abdominal injuries. Injury to the major pancreatic duct, injury to other abdominal organs, or vascular injuries are the most important prognostic variables [6]. The presence of other solid organ lesions can raise mortality up to 10–20% [7]. On the other hand, the presence of isolated pancreatic trauma is rare, accounting for <10% of the cases [8].

This example further emphasizes the significance of referring a complex pancreatic trauma patient to a tertiary HPB unit as soon as possible to avoid late management in a much more severely ill patient. The patient returned to the referring hospital ~24 hours after his first admission due to increased upper abdominal pain. Following the acquisition of CT images, he was quickly referred to our HPB center, where he received immediate and suitable final treatment.

CT may not be conclusive in some cases regarding main pancreatic duct disruption (MPD) [3, 9], which is the determining factor for surgical treatment.

Our patient had an isolated pancreatic trauma and the initial CT scan revealed some signs of a complete distal pancreatic transection, including a body laceration throughout the parenchyma and a large fluid collection anterior to the pancreas and communicating with the transection. These CT findings suggested that the patient had a pancreatic injury of AAST grade III. MRI could provide us with more information about possible ductal injury [10].

Endoscopic management has been described as a valuable option for isolated grade I and II pancreatic injuries in recent years [2, 4]. Endoscopic retrograde pancreatography (ERP) evaluates the MPD’s integrity and continuity, and if there is only a partial injury to the MPD and its continuity remains intact, a stent is placed for add-on therapy [11]. Initial ERP was not an option for our hemodynamically challenged patient. Several surgical procedures have been described as viable options for managing grade III pancreatic injuries. Because of the high risk of pancreatic fistula formation, which would exponentially increase morbidity, parenchyma-preserving surgical procedures such as drainage only, pancreaticojejunostomy, or pancreaticogastrostomy were not considered. Distal pancreatectomy has been accepted as the best treatment option for major distal pancreas lacerations [8]. Damage control surgery, including drainage only at the initial laparotomy, has been shown to be the best solution in critically ill patients [2, 8]. A delayed resection will be performed during a subsequent intervention when the patient is no longer hemodynamically challenged [12]. In this case, the patient was on a low dose of vasopressor drugs to maintain mean arterial pressure and there were no other organ lesions at the exploratory laparotomy, leading to the decision to proceed with the pancreatic resection. Because the patient was ill, we decided to perform an en bloc splenectomy. To preserve splenic function, spleen-preserving distal pancreatectomy has been described as a viable option in the treatment of grade III pancreatic injury [6, 13, 14] particularly in younger patients. Because it is a time-consuming procedure, spleen-preserving distal pancreatectomy is only possible in patients who are not severely injured. This surgical option was not appropriate for our patient, who also had a large accessory spleen that was kept in place. Pancreatic fistula is the most common complication after surgical resection for pancreatic trauma, with rates ranging from 38 to 75% [4, 15]. Pancreatic injury grade IV and pre-operative peritonitis signs are the main risk factors for pancreatic fistula [15]. In this particular case, the patient presented to surgery with an acute abdomen. He had a pancreatic fistula (grade B) and later a fluid collection, both of which were successfully managed conservatively. Six weeks after the surgery, the patient is healthy and asymptomatic.

## Conclusion

Isolated pancreatic trauma is extremely rare and has a high morbidity. In the presence of vague but persistent upper abdominal pain, clinicians must maintain a high level of suspicion for pancreatic injury. The presence of other intra-abdominal lesions, the hemodynamic status, and the integrity of the main pancreatic duct will all dictate patient management in the case of distal pancreatic trauma. Distal pancreatectomy with en bloc splenectomy remains the best surgical option for hemodynamically challenged patients who are not critically ill.
